# Impact of droughts on water provision in managed alpine grasslands in two climatically different regions of the Alps

**DOI:** 10.1002/eco.1607

**Published:** 2015-02-03

**Authors:** Georg Leitinger, Romed Ruggenthaler, Albin Hammerle, Sandra Lavorel, Uta Schirpke, Jean‐Christophe Clement, Pénélope Lamarque, Nikolaus Obojes, Ulrike Tappeiner

**Affiliations:** ^1^ Institute of EcologyUniversity of InnsbruckInnsbruckAustria; ^2^ Institute for Alpine EnvironmentEuropean Academy of Bolzano/BozenBolzano/BozenItaly; ^3^ Laboratoire d'Ecologie AlpineUniversité Joseph FourierGrenobleFrance

**Keywords:** climate change, deep percolation, evaporation, HILLFLOW model, spatial analyses, transpiration

## Abstract

This study analyzes the impact of droughts, compared with average climatic conditions, on the supporting ecosystem service *water provision* in sub‐watersheds in managed alpine grasslands in two climatically different regions of the Alps, Lautaret (French Alps) and Stubai (Austrian Alps). Soil moisture was modelled in the range of 0–0.3 m. At both sites, current patterns showed that the mean seasonal soil moisture was (1) near field capacity for grasslands with low management intensity and (2) below field capacity for grasslands with higher land‐use intensity. Soil moisture was significantly reduced by drought at both sites, with lower reductions at the drier Lautaret site. At the sub‐watershed scale, soil moisture spatial heterogeneity was reduced by drought. Under drought conditions, the evapotranspiration to precipitation ratios at Stubai was slightly higher than those at Lautaret, indicating a dominant ‘water spending’ strategy of plant communities. Regarding catchment water balance, deep seepage was reduced by drought at Stubai more strongly than at Lautaret. Hence, the observed ‘water spending’ strategy at Stubai might have negative consequences for downstream water users. Assessing the water provision service for alpine grasslands provided evidence that, under drought conditions, evapotranspiration was influenced not only by abiotic factors but also by the water‐use strategy of established vegetation. These results highlight the importance of ‘water‐use’ strategies in existing plant communities as predictors of the impacts of drought on water provision services and related ecosystem services at both the field and catchment scale. © 2015 The Authors. *Ecohydrology* published by John Wiley & Sons, Ltd.

## Introduction

Increased frequencies and intensities of droughts are extreme events that are projected to occur in terrestrial ecosystems by the end of the 21st century at the latest (Reichstein *et al*., 2013; Bahn *et al*., 2014). Given these expectations, the related changes in ecosystem services (ESs) (e.g. biomass and forage quality, carbon sequestration and water provision) are being studied intensively (Jentsch *et al*., 2011; Walter *et al*., 2012; Lamarque *et al*., 2014), as well as their effects on society, economy and the Earth system (Cerda *et al*., 1998; Vetter & Bond, 2012; Martínez‐Garza *et al*., 2013; Reichstein *et al*., 2013). A crucial step for the successful implementation of the concept of ES (Lamarque *et al*., 2011a) in regional resource management and policy is to quantify and map the provisioning of ESs (Seppelt *et al*. 2011; Burkhard *et al*. 2012). Quantification is necessary to evaluate the trade‐offs between ESs when making decisions (Egoh *et al*., 2008; Seppelt *et al*., 2011; Crossman *et al*., 2012). The most promising techniques for quantification, reported in the study of Martínez‐Harms and Balvanera (2012), are the following: (1) extrapolation of primary data to the analysed area, producing spatially explicit results by combining quantitative and qualitative aspects; however, careful interpretation of these results is needed because of the potential lack of representation of the stochastic, scale‐dependent and nonlinear nature of ecological processes, thereby producing a uniformity of error. This may occur when an average ES value is attributed to a distinct cartographical unit (i.e. land use/cover type) and (2) a combination of plot measurements and empirical geostatistical models to spatially model ESs provision. Detailed reviews on the studies can be found in the study of Martínez‐Harms and Balvanera (2012) and Nemec and Raudsepp‐Hearne (2013).

Nevertheless, depending on the nature of the ecosystem functions and processes, the use of a process‐based (e.g. physical) environmental model is occasionally obligatory to correctly reproduce spatial configurations and patterns (Eigenbrod *et al*., 2010). This is particularly true for hydrological ESs that are strongly affected by the changes in ecosystems due to global change (Beniston *et al*., 2007; Bangash *et al*., 2013; Crossman *et al*., 2013; Liu *et al*., 2013), for which topographical and physical characteristics and the related three‐dimensional movement of the water must be taken into account. Hydrological ES in mountain areas is based on complex interactions between topography, soil characteristics, vegetation and climate, all of which influence the runoff production (Wigmosta *et al*., 1994). These characteristics require high‐quality models for quantification. However, complex models propagate uncertainty based on two causes: (1) model uncertainty, i.e. uncertainty regarding the description of processes in the models and uncertainties due to the parameter interactions in more complex models and (2) multiplication of parameters, which results in additional sources of uncertainty.

In particular, mountainous areas are important target regions for the quantification of hydrological ESs due to their vital role as the water towers of the world (Viviroli *et al*., 2003; Messerli *et al*., 2004). Although only a quarter of the world's population lives in mountainous areas (Meybeck *et al*., 2001), more than half of it relies on water coming from the mountains (Beniston, 2006). Additionally, mountain areas are known to be more affected by extraordinary rainfall events than other landscapes (Serrano‐Muela *et al.*, 2013; Taguas *et al*., 2013; Wang *et al*., 2014). The future climate will involve, not only increased air temperatures but also more frequent and more intense extreme events (Seneviratne *et al*., 2012; Bahn *et al*., 2014; Köplin *et al*., 2014). Grasslands are primarily susceptible to drought events and less susceptible to other extremes (Reichstein *et al*., 2013). Drought affects water provision and consequently important ESs in managed grasslands such as forage production or forage quality (Zwicke *et al*., 2013, but refer to the study of Jentsch *et al*. (2011)). However, Benot *et al*. (2013a) found that the short‐term influence of management on plant diversity and biomass production was stronger than the influence of extreme summer weather for the upper valley of the Romanche River in the Central French Alps. Therefore, both climate change effects in the long term and distinct responses of grassland ecosystems to extreme events are expected because grassland ES is affected by both climate‐use and land‐use changes (Soussana & Luscher, 2007).

This study aimed to analyse the impacts of drought on the supporting **water provision** ES in managed mountain grasslands in two climatically different regions of the Alps. Stakeholder consultations in both regions revealed the importance of water provision for forage production, grazing and hydropower (Lamarque *et al*., 2011b). As indicators of ES water provision, stakeholders living in the French Alps stressed the importance of *soil moisture* affected by drier climatic conditions, while stakeholders from the study site in the Austrian Alps mentioned *water quantity* (i.e. hydropower) as being more important (Lamarque *et al*., 2011b). To address the future challenges for grassland management due to climate change, the impacts of seasonal droughts on *soil moisture* and *water quantity* based on highly probable precipitation scenarios (Beniston, 2006; Beniston, 2012; Strauss *et al*., 2013) were evaluated.

Our objectives were as follows:

- to implement a physically based, well‐calibrated hydrological model for alpine grassland sites at two climatically different study sites;
- to quantify the water provision service by modelling *soil moisture* and *water quantity* for the current land use/cover at both sites under current climate conditions;
- to evaluate the impacts of seasonal drought on *soil moisture* and *water quantity* at each of these sites given the current land‐use conditions

## Materials and Methods

### Study areas

**Figure 1:**
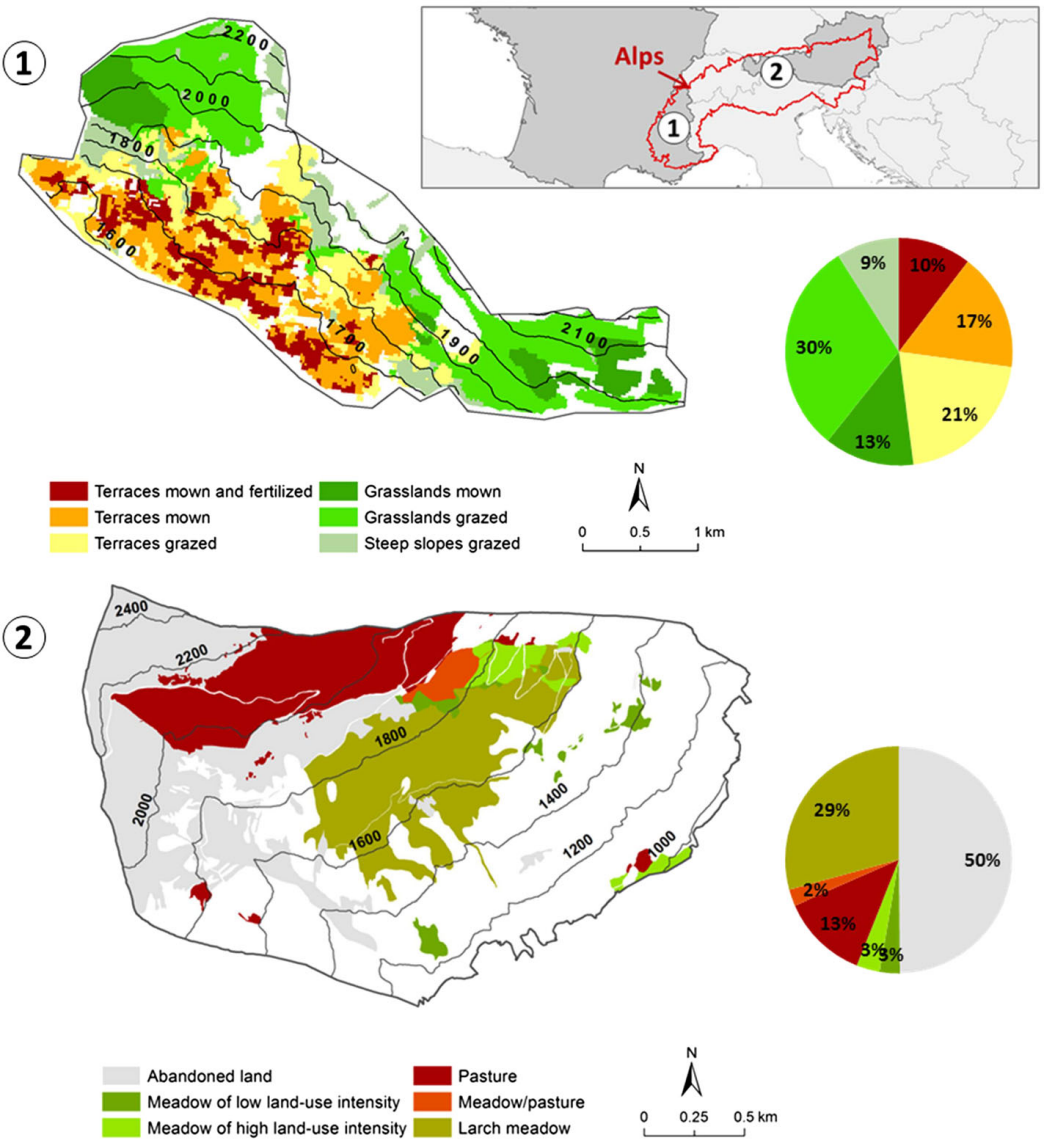
Location of the study areas in the Alps: site Lautaret in the Upper Romanche Valley in France (1); site Stubai in the Stubai Valley in Austria (2). The regions of the study areas depicted in white represent non‐grassland.

The experimental long‐term socio‐ecological research (LTSER) site **Lautaret** (**F**) (Lavorel *et al*., 2013) covers 12.92 km^2^ between 1650 m and 2500 m a.s.l. and is located on the south facing the slopes of the valley above the village of Villar d'Arène in the central French Alps (N45.04°, E6.34°; Figure 1). Lautaret is characterized by a subalpine climate with a strong continental influence due to a rain shadow effect deriving from dominant westerly winds. The mean annual air temperature is 5 °C at 1650 m a.s.l. and 3 °C at 2000 m a.s.l.; the mean annual precipitation is 956 mm. Most of the precipitation falls as snow during the winter, whereas *ca* 18% of the annual rainfall occurs during the summer. The current landscape is dominated by grassland ecosystems of the soil type *Cambisol*. These grasslands are still used by a small and active farming community centred around sheep and cattle rearing for lamb and steer production. At lower altitudes (e.g. 1650–2000 m a.s.l.), former arable fields have been abandoned and subsequently converted to terraced grasslands used for hay production or grazing. At midslope (e.g., 1800‐2200 m a.s.l.), ancient, never ploughed hay meadows are increasingly used for light summer grazing by sheep or cattle; a small fraction of these areas are no longer cut or grazed at all. The upper slopes (2200–2500 m a.s.l.) are grazed by transhumant flocks during the summer (Quetier *et al*., 2007).

The experimental site **Stubai** (**A**) is located at the LTSER site *Kaserstattalm* (Tappeiner *et al*., 2013) in the Stubai Valley in the Austrian Alps and covers 4.93 km^2^ between 970 and 2200 m a.s.l. (N47.13°, E11.33°; Figure 1). The mean annual precipitation ranges from 852 to 1097 mm at 970 and 1900 m a.s.l., respectively. The mean annual air temperature is 6.5 °C at 970 m a.s.l. and 3.0 °C at 1900 m a.s.l. The present grasslands are found on the soil type *Dystric* C*ambisol* and differ in management intensity. A tendency towards reduced management and less intensive grazing since the 1950s has resulted in a successive change of abandoned areas to shrublands and young forest stands, shaping the landscape structure of the Stubai Valley mountain region.

Further details on grassland types as well as relevant soil and vegetation characteristics can be found in Table 1. Please note that to allow comparison with other studies at our experimental sites, numbering of grassland types does not follow chronological order.

**Table 1:** Area of investigated grassland types for Lautaret (L) and Stubai (S) with soil hydrological properties at a depth of 0–0.3 m [field capacity (FC), gross pore volume (GPV), permanent wilting point (PWP)], evapotranspiration of grassland community May–October (ET) and vegetation type (data for Lautaret: Quetier *et al*. (2007), Obojes *et al*. (2014); data for Stubai: Obojes *et al*. (2014), unpublished measurements by the authors).

Grassland type	Description	Area	FC	PWP	ET	Plant community
		(ha)	(Vol%)	(Vol%)	(mm d^−1^)	
L1	Previously cultivated terraces (1550–1950 m), now manured and mown for hay	73.1	37	25	2.9–4.1	Mown and fertilized meadows dominated by *Dactylis glomerata*, and *Trisetetum flavescens*
L2	Previously cultivated terraces (1550–1950 m), now mown for hay but not manured	118.1	40	23	2.8–3.5	Mown meadows dominated by *B*. *erectus* and *Sesleria caeruela*
L3	Previously cultivated terraces (1550–1950 m), now unmown and grazed in spring and autumn	146.3	38	22	2.9–3.6	Pastures with low management intensity dominated by *B*. *erectus* and *S*. *caeruela*
L4	Never cultivated unterraced grasslands (1700–2000 m), currently mown for hay	90.0	37	22	3.0–3.6	Mown meadows dominated by *Festuca paniculata*, *Meum athamanticum*, and *Trifolium alpinum*
L5	Never cultivated unterraced grasslands (1700–2000 m), summer grazed	213.2	38	21	3.4–3.9	Pastures of low management intensity dominated by *F*. *paniculata* and *Festuca nigrescens*
L9	Steep and rocky slopes, grazed	63.0	37	20	2.9–3.6	Sloping pastures with very low management intensity dominated by *B erectus*, *S*. *caeruela* and several xeric species
S2	Abandoned land	113.0	40	20	3.8–6.9	Abandoned meadow dominated by *Luzula sylvatica*, *Geranium sylvaticum* and *Potentilla aurea*, increasing abundance of *Vaccinium sp*.
S3	Pasture	28.0	37	21	3.8–7.2	Pasture of high management intensity dominated by *A*. *vulgaris*, *L*. *hispidus*, and *R*. *acris*
S4	Meadow of low land‐use intensity (1100–1900 m), mown every 2 years, not manured	6.5	37	20	4.1–7.2	Mown meadows of low management intensity dominated by *Leontodon hispidus*, *Agrostis capillaris*, and *Plantago lanceolata*
S6	Meadow of high land‐use intensity (1600–1900 m), 1–2 mowings per year, manured	7.5	37	20	4.1–7.2	Mown and fertilized meadows of high management intensity dominated by *Alchemilla vulgaris*, *A*. *capillaris*, and *Ranunculus acris*
S8	Meadow/pasture	5.3	42	21	4.1–7.0	Mown meadow and pasture of low management intensity dominated by *A*. *vulgaris*, *A*. *capillaris*, and *R*. *acris*
S9	Larch meadow	66.3	40	20	3.6–6.7	Mown meadow of low management intensity with *Larix decidua* dominated by *L*. *hispidus*, *A*. *capillaris*, and *P*. *lanceolata*

### Hydrological model HILLFLOW

Given that both investigated study areas are not closed catchments, hydrological modelling was performed with the 3D version of the hillslope model HILLFLOW developed by Axel Bronstert (University of Potsdam) and Erich Plate (University of Karlsruhe). HILLFLOW is described by its authors as a physically based, distributed hydrological model at the hillslope and small catchment scale, although there are some sub‐models containing empirical approaches (e.g. Mualem–van Genuchten model for unsaturated water flow). Detailed information can be found, e.g. in the study of Bronstert (1999), Bronstert and Bardossy (1999) and Bronstert and Plate (1997).

Hydrological modelling and model calibration were performed based on land‐use/cover (LUC) maps and a comprehensive database on (micro) climatic variables, botanical compositions, plant functional traits, soil characteristics and a range of ecosystem properties (e.g. evapotranspiration, biomass production, litter decomposition, nitrogen stocks and fluxes, etc.) of more than 60 permanent plots distributed across different LUC types and altitudes (Lavorel *et al*., 2013; Tappeiner *et al*., 2013). HILLFLOW 3D has a grid‐based discretization with quadratic‐shaped, constant‐sized grids (Bronstert, 1995). Based on the available resolution of digital elevation models and LUC‐maps, the grid size was 75 m for Lautaret and 50 m for Stubai. As input data for each cell, parameters regarding the existing soil type (e.g. *soil texture*, *saturated water content*, *field capacity*, *residual soil water content*, *saturated hydraulic conductivity*, *soil depth* and *macropores*), vegetation type (e.g. *root depth*, *evapotranspiration* and *canopy interception*), topography (e.g. *slope* and *altitude*) and climate (e.g. *precipitation*) were required for the existing grassland types (Table 1). Among these parameters, *Mualem–van Genuchten parameters* (*α*, *n*), *saturated water content*, *saturated hydraulic conductivity*, *canopy interception* and *proportion of macropores* were estimated from available soil moisture observations. Drought effects were modelled by reducing the long‐term average (e.g. *normal*) precipitation (daily temporal resolution, Table 2). At Lautaret, the simulation of drought conditions was based on reducing the long‐term average (*normal*) precipitation (1970–2000) of a first drought in the spring and a second one in summer (refer to the study of Lamarque *et al*. (2013)). The selected precipitation scheme for Lautaret reflects the possible scenarios according to Beniston (2006), given that Mediterranean‐influenced areas will be faced with more extensive droughts throughout the vegetation period (refer to the study of Benot *et al*. (2013a)). At Stubai, the daily precipitation sum of the long‐term average (*normal*) from the years 1990–2010 was reduced in relative numbers to match the total precipitation in the year 2003 (e.g. a very dry summer). According to the studies of Strauss *et al*. (2013), Beniston (2006), and Beniston (2012), more frequent summer droughts, as in the year 2003 (ZAMG, 2014), are the most likely scenario in the coming 30 to 50 years. Selected precipitation regimes (e.g. *normal* and *dry*) were assumed to be homogenously distributed throughout each site. The modelling period was from May to September, covering the growing seasons at both sites. Thirty days prior to the modelled period were included as a ‘model wind up’ period to establish realistic soil water conditions.

**Table 2:** Monthly and seasonal (vegetation season) precipitation [mm] for the normal and dry scenario at Lautaret and the Stubai Valley.

Precipitation [mm]	Lautaret	Lautaret	Stubai Valley	Stubai Valley
	normal	dry	normal	dry
May	65.9	0.0	89.0	40.8
June	73.4	36.7	130.0	52.9
July	62.0	0.0	154.3	43.0
August	76.4	38.2	152.3	40.6
September	77.4	77.4	106.0	50.0
Sum	355.1	152.3	631.6	227.3

For model calibration at Lautaret, soil moisture data at a depth of 0.1 m (sensor type: Echo‐probe, Decagon Devices Inc., USA) was used. For Stubai, model calibration was performed using soil moisture data at depths of 0.05 m and 0.15 m (sensor type: ThetaProbe, Delta‐T Devices Ltd., UK). The calibration period was set from the beginning of May until the end of September, including a ‘model wind up’ period of 1 month, covering the moist conditions after the snow melt as well as droughts during the summer months. Thus, plausible parameter estimation over the entire realistic soil moisture range can be ensured (Gan *et al*., 1997). To calibrate the model, a least squares optimization was applied. In addition to standard regression parameters, model evaluation statistics for calibrated grassland types included Nash–Sutcliffe efficiency (NSE), percent BIAS, root mean square error (RMSE) and the RMSE observations standard deviation ratio (RSR). A detailed description of each statistical parameter is given by Moriasi *et al*. (2007). To evaluate the goodness of fit, the NSE of each calibration was classified according to the scheme from the study of Moriasi *et al*. (2007) (0.75 < NSE ≤ 1.00 very good; 0.65 < NSE < 0.75 good; 0.50 < NSE < 0.65 satisfactory; NSE ≤ 0.50 unsatisfactory).

For grasslands without available soil moisture data, soil parameters were taken from the calibrated grassland type, which was most likely representative, based on available soil data: L3(calibrated) = L9; S2(calibrated) = S9; S8(calibrated) = S3,S4.

## Results

### Parameterization and calibration

The results confirmed the applicability of the calibrated models, classifying five out of eight as very good (Table 3). The only exception was L5, which had a NSE coefficient of 0.48 and underestimated the soil moisture content. However, as indicated by a RMSE of 1.67, differences from the measured soil moisture content were low.

**Table 3:** Model evaluation statistics for calibrated grassland types. Statistics are standard regression parameters [slope, offset and coefficient of determination (R^2^)], Nash–Sutcliffe efficiency (NSE), percent BIAS (PBIAS), root mean square error (RMSE) and RMSE observations standard deviation ratio (RSR). Model performance was classified according to Moriasi *et al*. (2007) (+++ very good, ++ good, + satisfactory, − unsatisfactory) using NSE and RSR.

	Model evaluation statistics								
	Standard regression								
Grassland type	Slope	Offset	R^2^	NSE		PBIAS	RMSE	RSR	
L1	0.87	3.81	0.84	0.81	+++	1.86	1.61	0.44	+++
L2	0.95	2.01	0.81	0.79	+++	−0.14	1.46	0.46	+++
L3	1.04	−1.07	0.82	0.76	+++	−1.08	2.24	0.49	+++
L4	0.71	10.42	0.69	0.54	+	3.52	2.47	0.68	+
L5	0.71	12.08	0.66	0.48	−	2.19	1.67	0.72	−
S2	0.52	17.32	0.76	0.66	++	−2.01	2.55	0.58	++
S6	0.78	8.23	0.92	0.89	+++	−1.03	1.29	0.33	+++
S8	0.74	11.38	0.85	0.84	+++	−0.09	1.47	0.40	+++

**Figure 2:**
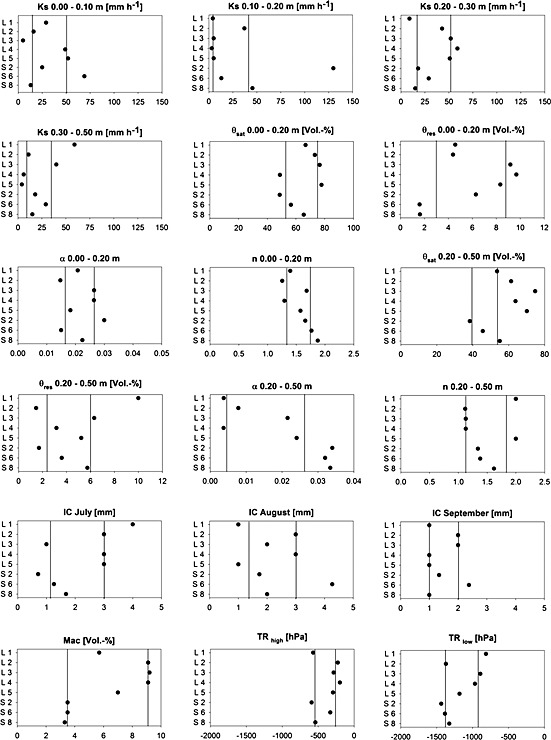
Parameter estimates from model calibration: *Ks* = saturated hydraulic conductivity; *Ɵsat* = saturated water content; *Ɵres* = residual soil water content; *α*, *n* = Mualem–van Genuchten parameters; *IC* = canopy interception (water); *Mac* = proportion of macropores; *TR* = transpiration reduction (at high/low potential rates). Black vertical lines mark the interquartile range of all grassland types.

Parameter estimates revealed the differences in physical and hydrological soil parameters between Stubai (A) and Lautaret (F) (Figure 2). Regarding soil hydrological parameters, saturated conductivity (*Ks*) and residual soil water content (*Ɵres*) showed similar values. However, saturated soil water content (*Ɵsat*) was slightly higher at the Lautaret site. This fact was corroborated by the calibrated Mualem–van Genuchten parameters *α* and *n* having lower values at Lautaret indicating higher clay content (Hartge & Horn, 1999). The parameter TR described the soil water tension (e.g. suction head) at which potential transpiration is reduced by plants. This process is mainly regulated by the stomatal control of the plants and is therefore highly related to species composition. In more detail, the HILLFLOW model required two different values for TR varying with different rates of potential transpiration (*TR*): (1) *TR*_high_ for high potential transpiration rates (>5 mm d^−1^) and (2) *TR*_low_ for low potential transpiration rates (<1 mm d^−1^). For both TR_high_ and TR_low_, the best parameter estimates indicated a reduction in the potential transpiration rate at higher soil water tension levels at Lautaret compared with Stubai. Although saturated water contents as well as clay content (as a proxy for soil water retention capacity) were higher at Lautaret, parameterization of *TR*_high_ and *TR*_low_ indicated that plants were closing their stomata earlier to reduce transpiration rates and to save available water in the root zone.

### Soil moisture

Soil moisture was modelled in the range of 0–0.3 m to reproduce the impact of drought on the main rooting depth of grassland communities (Tasser & Tappeiner, 2005). At Lautaret, grasslands with higher management intensities (L1 > L2 > L3 on terraces; L4 > L5 on unterraced grassland) were prone to lower soil moisture under both *normal* and *dry* conditions (Table 4). Unterraced grassland showed generally higher soil moisture values than terraced grassland but also exhibited the strongest reductions with drought. At Stubai, no distinct correlation between management intensity and soil moisture was observed. However, grasslands with the highest management intensities (S6, S8) were more strongly affected by drought. In contrast, pastures showed the lowest negative impact of drought (Table 4). The mean soil moisture at drought was generally lower, and the relative reduction of soil moisture by drought was generally higher at Stubai compared with Lautaret for land‐use types with similar management intensity (i.e. S6 vs L1; S4 vs L2 and L4; S3 vs L3 and L5; S8 vs L4; S9 vs L4; Table 4).

**Table 4:** Modelled mean soil moisture (Vol.−%) and standard deviation for different grassland types for normal (n) and dry (d) years; difference (Δ n–d) in absolute values (Vol.−%) and relative numbers (%) at a depth of 0–0.3 m.

Site‐ID	Description	Normal (n) year	Dry (d) year	Δ n–d (%)
L1	Previously cultivated terraces (1550–1950 m), now manured and mown	33.63 (±1.4)	26.01 (±0.6)	−7.62 (−22.7%)
		
L2	Previously cultivated terraces (1550–1950 m), now mown but not manured	35.76 (±1.8)	26.01 (±1)	−9.75 (−27.3%)
		
L3	Previously cultivated terraces (1550–1950 m), now unmown and grazed in spring and autumn	36.56 (±1.8)	26.22 (±1.3)	−10.34 (−28.3%)
		
L4	Never cultivated unterraced grasslands (1700–2000 m), currently mown	39.49 (±1.3)	28.28 (±1)	−11.21 (−28.4%)
		
L5	Never cultivated unterraced grasslands (1700–2000 m), summer grazed	43.21 (±2.0)	27.48 (±1.9)	−15.73 (−36.4%)
		
L9	Steep and rocky slopes, grazed	37.75 (±2)	26.91 (±1.9)	−10.84 (−28.7%)
		
S2	Abandoned land	33.03 (±0.6)	20.27 (±1.3)	−12.76 (−38.6%)
		
S4	Meadow of low land‐use intensity (mowing every 2 years)	33.00 (±1.4)	21.34 (±2.4)	−11.66 (−35.3%)
		
S6	Meadow of high land‐use intensity (1–2 mowings per year)	35.04 (±1.3)	20.19 (±1.3)	−14.85 (−42.4%)
		
S3	Pasture	29.58 (±1.6)	21.85 (±1.1)	−7.73 (−26.1%)
		
S8	Meadow/pasture	39.42 (±2.6)	22.35 (±1.1)	−17.07 (−43.3%)
		
S9	Larch meadow	32.92 (±0.4)	20.55 (±1.1)	−12.37 (−37.6%)
		

**Figure 3:**
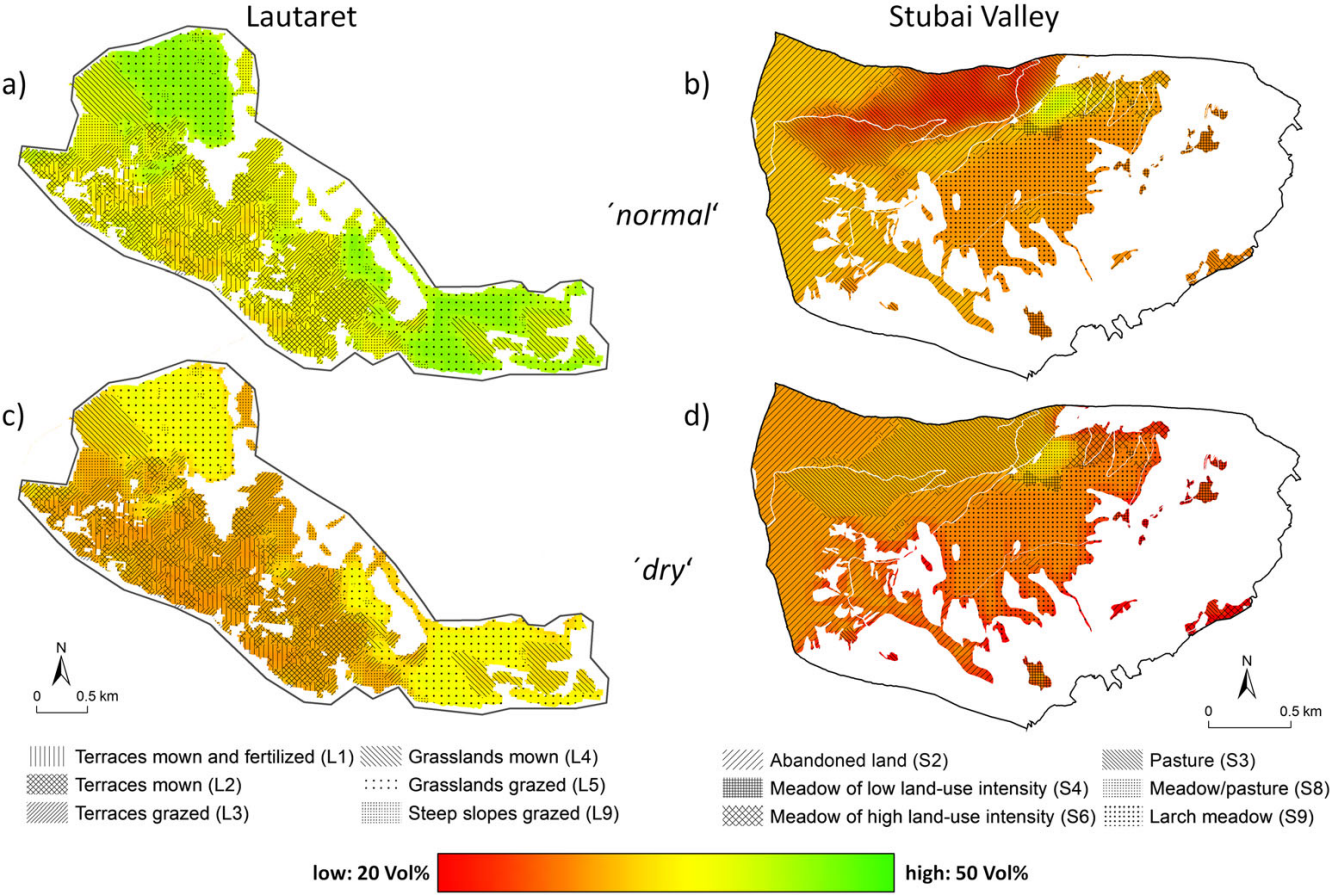
Spatial soil moisture distribution at a depth of 0–0.3 m under ‘normal’ (a, b) and ‘dry’(c, d) conditions at Lautaret and Stubai.

The soil moisture patterns in Figure 3 are a graphic representation of the results presented in Table 4. Regarding the differences across the grassland types under current (i.e. *normal*) conditions, greenish areas mark the mean seasonal soil moisture close to field capacity (cf., Table 1, Table 4). At both sites, this category was attributed to grasslands with low management intensity (i.e. unterraced, no fertilisation, not heavily grazed, max. 1 cut per year). On the other hand, grasslands of higher land‐use intensity (i.e. terraced grassland at Lautaret and meadows of high land‐use intensities as well as larch meadows at Stubai) revealed the mean soil moisture below field capacity, indicated in yellow and orange. As the soil types present (*Dystric Cambisol*, *Cambisol*) did not vary significantly regarding soil hydrological properties (Table 1), soil moisture is reduced by varying the biotic responses (i.e. ET). Even under ‘normal’ conditions, soil moisture at the most productive grassland sites was below its optimum for plant growth. Pastures (S3) at Stubai showed the lowest mean soil moisture values caused by simultaneously high ET and low management intensity (i.e. low biomass removal) – a unique combination compared with other grassland types.

Under ‘dry’ conditions, both study sites showed clearly reduced soil moisture for each grassland type, but the effect was smaller at Lautaret. Current differences between grasslands were almost levelled out by drought effects at Stubai. At Lautaret, unterraced grassland types (L4, L5, L9) showed clearly higher soil moisture values compared with terraced grassland of higher land‐use intensities. Generally, spatial heterogeneity of soil moisture was reduced under ‘dry’ conditions.

### Water quantity

**Figure 4:**
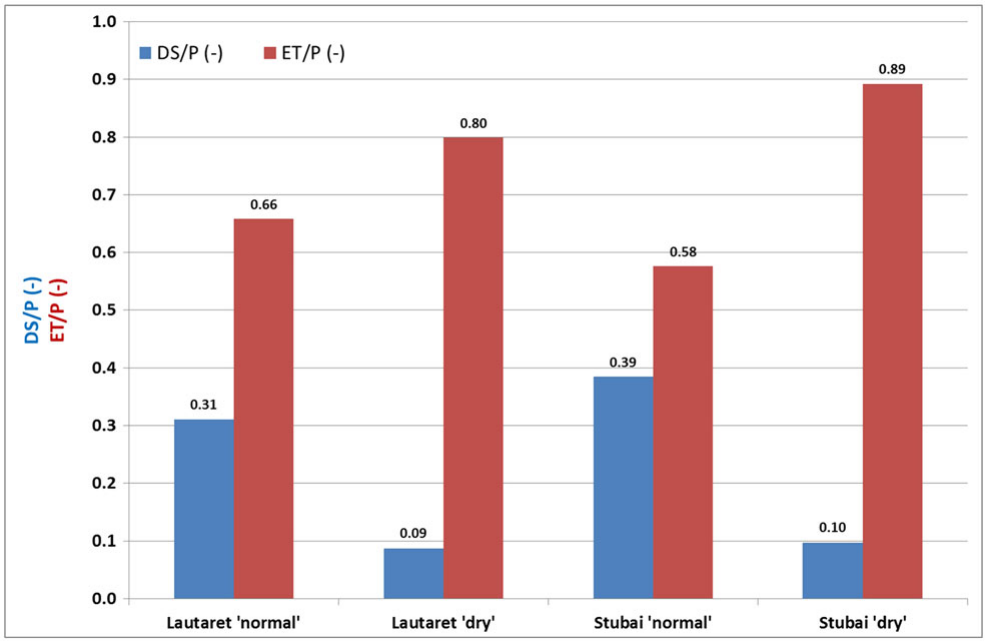
Relating deep seepage and evapotranspiration to precipitation for evaluating water balance components under ‘normal’ and ‘dry’ conditions at Lautaret and Stubai.

The following results refer to the totals for the modelling period May to September, unless otherwise specified. In absolute numbers, under ‘normal’ conditions, ET made up 234 mm and 366 mm at Lautaret and Stubai, respectively. Under ‘dry’ conditions, ET was reduced to 122 mm and 202 mm at Lautaret and Stubai, respectively. Water quantity [i.e. deep seepage (DS)] was evaluated at the catchment scale and resulted from the HILLFLOW model output. Relating DS to precipitation (P) and ET to P showed the differences in the water balance between the drier site, Lautaret, and the more humid site, Stubai. The model estimated that under ‘normal’ conditions, 31% (110 mm) and 39% (246 mm) of precipitation drained as DS water (Figure 4) at Lautaret and Stubai, respectively. Under ‘dry’ conditions, DS was reduced to 19 mm and 26 mm at Lautaret and Stubai, respectively. While higher DS/P values at Stubai indicate more humid conditions than at Lautaret, higher ET/P values indicate drier conditions, which were only observed for Lautaret under ‘normal’ conditions. Under ‘dry’ conditions, ET/P ratios at Stubai were slightly higher, indicating a ‘water spending’ strategy.

## Discussion

Assessing the water provision service for alpine grasslands at two climatically different regions across the Alps provided evidence that, under drought conditions, ET is influenced not only by abiotic factors (i.e. soil and climate) but also by the water‐use strategy of the vegetation present. This influence was obvious at the drier site, Lautaret, where the mean soil moisture under drought conditions was higher than at the more humid site, Stubai. Given soil depths of at least 0.3 m (lowest on terraced grassland, Lautaret) (Robson *et al*., 2007; Leitinger *et al*., 2010), thereby not limiting the main rooting depth of the investigated plant communities (e.g. Tasser and Tappeiner (2005)), it must be noted that the depletion of soil moisture mainly depended on potential ET rates defined for every existing vegetation type. To overcome a possible bias by overestimating ET, our parameterization and calibration procedure covered wet and dry periods as well as all parameters that trigger ET reduction due to lower soil moisture and general climatic conditions (i.e. clear vs overcast skies). The amount of soil moisture depletion during the modelled drought periods was within the range covered by the period of model calibration, and the results are therefore considered highly reliable. The performance of the model was at least satisfactory for the seven out of eight investigated grassland types (Table 3), while only L5 was not calibrated satisfactorily. This finding was attributed to the high litter accumulation in this grassland type (Gross *et al*., 2007), which affected the soil moisture in two counteracting ways that are difficult to quantify. Litter layers may both reduce soil evaporation and increase interception of rainfall (Gross *et al*. 2008). Nevertheless, given the RMSE of 1.67 Vol% (RMSE observations standard deviation ratio of 0.72, Table 3), the calibration of L5 can still be considered suitable for modelling the soil moisture dynamics on a daily basis.

In general, modelled soil moisture was consistent with the observations from the study of Gross *et al*. (2008) in 12 plots at 0–15 cm depth for Lautaret in the ‘normal’ precipitation scheme, whereas the highest soil moisture throughout the growing season was observed on unterraced grasslands (L4, L5) and the driest soils were on mown terraces (L1, L2), which are shallower and rockier (Robson *et al*., 2007). At Stubai, Leitinger *et al*. (2010) found the mean soil moisture values at a depth of 0–0.2 m for the period from 23 May 2007 to 02 October 2007 (752 mm of precipitation) of 39.44°Vol% (±6.6) and 33.62°Vol% (±9.2) for abandoned land (S2) and pasture (S3), respectively. Given the lower soil depth of observation and higher precipitation (i.e. 752 mm compared with the long‐term average and model input of 631 mm between May and September), these results can be considered comparable to the modelled mean soil moisture from this study (cf., Table 4); this is particularly true with regard to the generally higher mean soil moisture at abandoned lands compared with pastures. Surprisingly, under ‘normal’ conditions, no clear differences in soil moisture patterns between the two sites were found. This result suggests that, while the mean soil moisture throughout the season did not differ significantly between the two sites, the stronger soil moisture dynamics reported by Obojes *et al*. (2014) at the drier Lautaret site promoted the establishment of plant communities adapted to generally drier conditions, as shown by lower Ellenberg moisture (F) values.

### Drought impact on soil moisture

Under ‘dry’ conditions, water shortage leads to lower stomatal conductance and reduced photosynthesis, as well as reduced biomass production (Chaves *et al*., 2002). Regarding the overall effects of drought at each site, Lautaret revealed higher soil moisture values than Stubai even though Lautaret is characterized by a drier climate where drought would be expected to have a stronger effect on soil moisture. In other words, the depletion of soil moisture under dry conditions was lower at Lautaret than at Stubai, which indicates different plant feedbacks driven by different climatic conditions (i.e. either precipitation amount or frequency). In general, drought leads to a more uniform soil moisture distribution in both catchments (Figure 3), which reflects a decoupling from the influence of vegetation given by soil moisture conditions close to the permanent wilting point (PWP).

The impact of drought at Lautaret was decreased soil moisture, ranging from −22.7% to −36.4% (Table 4), which is in line with the findings from the study of Benot *et al*. (2013b) who observed a decrease in the mean soil moisture of between −28% and −32% in a subalpine grassland site at Lautaret during a simulated extreme drought in July and August (−80% precipitation). The remaining soil moisture was above the PWP (cf., Table 1 and Table 4), indicating that plant communities were following a ‘water saving’ strategy to avoid structural or physiological damage. This is corroborated by the findings from the study of Benot *et al*. (2013a) at Lautaret, where community structure of a subalpine grassland was not affected after 2 years of consecutive summer drought due to well‐adapted plant communities. On the contrary, at Stubai, soil moisture decreased between −26.3% and −43.3%, reaching the PWP for most grassland types (cf., Table 1 and Table 4). Schmitt *et al*. (2010) and Brilli *et al*. (2011) report that, for differently managed grassland ecosystems at Stubai, low soil moisture did not lead to strong reductions in the net ecosystem CO_2_ and H_2_O exchange. Experiments conducted by Brilli *et al*. (2011) confirmed that at least the dominant grassland plant species were not sensitive to long drought periods until very low soil moisture was reached (<0.10 m^3^ m^‐3^).

Plant communities at Stubai more efficiently deplete soil moisture by acting as ‘water spending’ plants (Levitt, 1980; Moreno‐Gutierrez *et al*., 2012), which is reflected by higher estimates for the parameters *TR*_low_ and higher *TR*_high_ (Figure 2) at Lautaret compared with Stubai. This means that, at Lautaret, plants reduce ET earlier than at Stubai by closing their stomata (Lavorel, unpublished observation). With respect to drought periods, it can be concluded that drought would affect the soil moisture of grassland ecosystems at Stubai more strongly than at Lautaret unless physiological adaptations or shifts in plant communities are taking place. In regard to that, it remains debatable whether plants can initially adapt to drought physiologically by changing their water‐use strategy from ‘water spending’ to ‘water saving’ until such time as a shift to a more adapted plant community takes place (Jentsch *et al*., 2011; Reyer *et al*., 2013).

### Drought impact on water quantity

In the catchment water balance, DS is more strongly reduced by drought at Stubai than at Lautaret in both relative and absolute numbers (Figure 4). This study therefore confirms that the observed ‘water spending’ vegetation strategy at Stubai may have negative consequences for downstream water users (Brilli *et al*., 2011), i.e. reduced *water quantity*. At Lautaret, Obojes *et al*. (2014) found slightly higher precipitation rates of 2.75 mm d^−1^ compared with 2.3 mm d^−1^ in our study, as well as a DS values of 0.77 mm d^−1^ (±0.22) and ET rates of 2.07 mm d^−1^ (±0.24) under ‘normal’ conditions. Given the modelling period of 153 days, the findings from our study correspond to approximately 0.7 mm d^−1^ and 1.5 mm d^−1^ of DS and ET, respectively. At Stubai, a DS value of 1.6 mm d^−1^ and ET value of 2.4 mm d^−1^ at *P* = 4.1 mm d^−1^ were found. Obojes *et al*. (2014) report a DS of 3.04 mm d^−1^ (±0.39) and ET of 2.15 mm d^−1^ (±0.32), which is again realistic given the higher *P* rates of 5.17 mm d^−1^ (±0.21) leading to more DS, in contrast with the higher ET in a non‐water limited grassland ecosystem (Wieser *et al*., 2008). ET values under ‘normal’ conditions at Stubai also correspond to the results from the study Wieser *et al*. (2008) who compared the experimental data from 16 grassland sites in the Austrian Alps between 580 and 2550 m a.s.l. and estimated the mean ET rates of 2.2 mm d^−1^. They further stated that during 2001–2006, annual ET/P values ranged from 0.53 (2002) to 0.91 (2006), while the fraction of precipitation evaporating to the atmosphere increased with decreasing precipitation. Wieser *et al*. (2008) state, in this context, that even during droughts in which up to 90% of P evaporates, these systems are not faced with water stress. For the investigated grassland sites, they also specified that the limitations of ET by closing stomata due to low soil moisture played a minor role, which is again in line with our findings concerning ‘dry’ conditions at Stubai.

Della Chiesa *et al*. (2014) evaluated the changes in grassland hydrological cycling along an elevation gradient in the Alps. Their results indicated the water stress conditions for vegetation in each year at lower altitude (1000 m a.s.l.) with a mean annual temperature of 8.1–9.5 °C and annual precipitation of 580 to 636 mm. At medium altitudes (1500 m a.s.l., mean temperature of 5.3–7.1 °C, precipitation of 632–686 mm), only a warmer and drier year caused drought, whereas no water stress was observed at highest altitude (2000 m a.s.l., mean temperature of 2.5–3.5 °C, precipitation of 620–706 mm). While the annual precipitation in the study of Della Chiesa *et al*. (2014) was lower than for both sites in this study, temperatures and according ET were comparable. Given the general climatic conditions at Lautaret and Stubai, the results from Della Chiesa *et al*. (2014) support the findings from our modelling study; that is, under ‘normal’ conditions, generally no drought effect due to low soil moisture occurred. They also modelled the effects of a precipitation change of 30% on ET and showed a slight reduction in ET at middle elevations and no change at high elevation. This corroborates our findings that well‐adapted plant communities can easily cope with the reductions in precipitation without strongly affecting ET.

The ET/P ratios of 0.66 at Lautaret and 0.58 at Stubai under ‘normal’ conditions were within the range of values reported by Wieser *et al*. (2008), collected for 16 grassland sites. They reported decreasing ET/P values from 0.7 to 0.1 with increasing P and higher ET/P values in the drier inner Alpine region compared with the more humid parts of the Alps. Nevertheless, the modelled soil moisture gave no hint that water limitation took place at both sites. This fact is in line with findings from the study of Everson (2001) who observed ET/P values of 0.44–0.56 in wetter and 0.64–0.69 in drier years in montane grasslands of South Africa that they classified as being not limited by soil moisture. In comparison, Gu *et al*. (2008) found ET/P values of 0.6 at a meadow on the Qinghai–Tibetan Plateau that was then classified as water‐limited. The modelled ET/P values in this study under ‘dry’ conditions revealed higher ET/P values for the Stubai site (0.89), indicating stronger water limitation than at Lautaret (0.80). At Stubai, at least the dominant plant species act as ‘water spending’ plants, maintaining maximum transpiration rates despite strong reductions in soil moisture. Again, we would like to note that Brilli *et al*. (2011) showed in laboratory experiments that plant species at Stubai did not regulate stomatal conductance until soil moisture reached the PWP. Our study therefore confirms that the impacts of drought on water provision services can only be evaluated by taking water‐use strategies of the dominant plant species into account. This fact will gain additional importance in a future climate where increasing air temperatures foster higher evapotranspiration rates and the main factor for runoff formation changes throughout ecosystems (Liu *et al*., 2014; Olang *et al*., 2014).

### Ecosystem service ‘water provision’

Based on the study of Lamarque *et al*. (2011b) on stakeholder perceptions of grassland ESs, eight ESs were considered to be of high importance for local farmers at the two sites, Lautaret and Stubai: (1) soil stability, (2) soil fertility, (3) water provision, (4) water quality, (5) forage quality, (6) forage quantity, (7) aesthetic value, and (8) carbon storage. In the following, we discuss the impacts of changes in water provision (i.e. *soil moisture* and *water quantity*) on the other relevant ESs with a special focus on the water‐related services.

In the short term, *soil fertility* will decrease, and *water quality* will increase as drought conditions increase soil mineral nitrogen and nitrogen mineralization and reduce leaching losses (Bloor & Bardgett, 2012). The most obvious impacts are implications for plant growth and tissue quality, leading to changes in *forage quality* and *forage quantity*. *Forage quality* would be slightly reduced because of lower tissue water and nitrogen content (Quetier *et al*., 2007; Benot *et al*., 2013a). *Forage quantity* would be lower because of the significantly lower evapotranspiration of plants (De Boeck *et al*., 2011; Schirpke *et al*., 2013a).

Longer‐term effects are associated with species turnover towards more parsimonious water management strategies. Drought will increase the root/shoot ratio (Chaves *et al*., 2002) and consequently *soil stability* by increasing rooting density (Tasser *et al*., 2003). Additionally, drought leads to an increasing abundance of shrubs or tussock grasses with a ‘water saving’ strategy, which significantly reduces snow gliding and related erosion risk because of the growth form (Tasser *et al*., 2003; Leitinger *et al*., 2008). *Forage quality* would be further reduced because of a decline of forbs and increase in shrubs or grasses (Harte *et al*., 2006). Management decisions due to recurrent droughts and accompanied disadvantages for agronomy would lead to a decrease in the *aesthetic value* due to abandonment (Schirpke *et al*., 2013b). *Carbon storage* might be reduced because of reduced vegetation productivity. However, lagged and legacy effects as well as other mechanisms of drought are complex and intertwined and act synergistically or antagonistically (Reichstein *et al*., 2013). Additionally, our evaluation of impacts of drought on ESs is based on existing farming systems. However, studies already report an adaptation to climate change rather than a stop of farm activity (Bindi and Olesen, 2011; Lamarque *et al*., 2013; Varadan and Kumar, 2014), and promising techniques to design adaptation strategies at farming level have developed (Schaap *et al*., 2013, among others). Hence, the impact of drought on a specific ES or even bundled ES cannot be ascertained with certainty nor is it generalizable for managed alpine grasslands. However, by taking the results of this study in two climatically different regions of the Alps into account, the impacts of drought on numerous ES can be determined as long as preconditions concerning the ‘water‐use’ strategy of existing plant communities are taken into account.

## Conclusions

Our modelling analysis of the impact of droughts on the supporting ES *water provision* in sub‐watersheds dominated by managed alpine grasslands in two climatically different regions of the Alps revealed common responses of decreasing soil moisture and DS. However, these reductions were lower in the generally drier French region because of a stronger ‘water saving’ strategy of plant communities. The expected impacts of drought on water provision services can only be evaluated by considering water‐use strategies of the dominant plant species. Thus, whether more frequent droughts will lead to a shift to better adapted plant communities at the more humid Austrian region remains debatable and depends on whether and to which extent plants can initially adapt to drought physiologically by changing their water‐use strategy.
